# Supplementary material for: Identifying long-term periodic cycles and memories of collective emotion in online social media
Source: PLoS One. 2019 Mar 21;14(3):e0213843. doi: 10.1371/journal.pone.0213843 (PMC6428299; doi:10.1371/journal.pone.0213843)
Supplement: S1 Appendix — Dictionary building procedureDates in which emotion decreased every yearEmotion dynamics before removing periodic cyclesEmotion dynamics after removing periodic cyclesResults of monthly and daily randomized seriesHistogram of differences of collective emotion. (PDF) [file pone.0213843.s001.pdf]

SI Appendix: Identifying long-term periodic cycles and memories of collective emotion in online social media

Yukie Sano, Hideki Takayasu, Shlomo Havlin and Misako Takayasu

1 Dictionary building procedure

The Following procedure were conducted to build our emotion dictionary.

1. Parse one word that best expresses the emotion from each POMS question item using the Japanese morphological analyzer, MeCab[1]
2. Confirm whether the parsed word expresses emotion by three independent people.
3. Add orthographical variants for words with the same pronunciation and meaning, but different spellings using UniDic[2]
4. Exclude very low frequency words. To keep adequate numbers of words in the emotion dictionary, we excluded the words that appeared fewer than five times per month as of October 2015, when no big events occurred
5. Modify very high frequency words that appear more than 1,000 times per day as of October 2015. If a word appears more than 1,000 times, add one or two new words to the original words using original question sentences. If the number is still over 1,000, add a period to the end of the phrase.
6. Adjust 20 to 25 words per emotion. If one emotion has more than 25 words, exclude low frequency words. If it still has fewer than 20 words, add synonyms using Japanese WordNet[3]. Note that there are 35 words for *Confusion* since there are many low frequency words as a whole.
7. Check whether only one word is dominant in each emotion. If one word occupies more than 20% in an emotion, modify the word by procedure 5.

[1] http://taku910.github.io/mecab/: Accessed December 6, 2016
[2] https://ja.osdn.net/projects/unidic/: Accessed December 6, 2016
[3] http://nlpwww.nict.go.jp/wn-ja/: Accessed December 6, 2016

8. Choose 20 weblog articles randomly per listed word and have three independent people check whether the words are consistent with each emotion. If more than half the weblog articles are not consistent with the emotion, exclude the words from the dictionary.

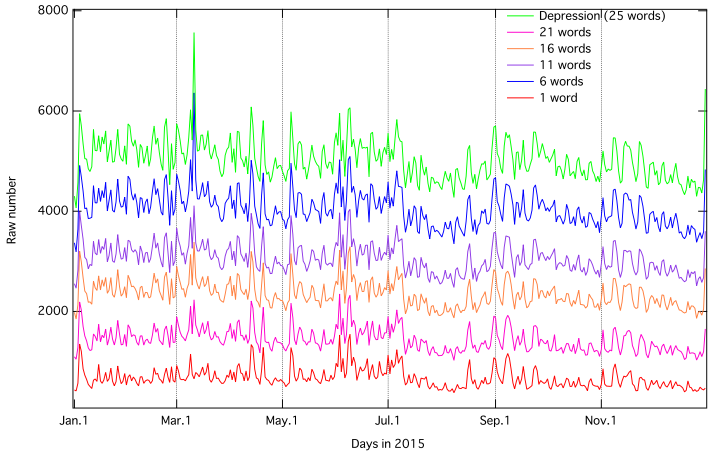

Figure 1: Example of the process of adding words to build an emotional time series for *Depression*. Time series adding five words in the raw number.

2 Dates in which emotion decreased every year

Table 1: **Dates in which emotion decreased less than** $90\% \pm 15\%$**.** Rates are calculated from the temporal average for the whole period expect for the 3.11 earthquake.

Date	Emotion	Rate (%)	Event
January 1	*Tension*	64.2	New Year's Day
	Depression	72.2	
	Anger	64.1	
	Vigor	89.0	
	Fatigue	60.1	
January 2	*Tension*	79.1	New Year's Day
	Anger	84.1	
January 3	*Tension*	87.3	New Year's Day
April 1	*Fatigue*	86.2	April fool
April 25	*Tension*	89.8	Golden Week (GW) holidays
April 28	*Tension*	89.6	
April 29	*Tension*	82.0	
April 30	*Tension*	86.0	
May 4	*Tension*	87.9	
May 5	*Tension*	87.6	
October 31	*Fatigue*	88.9	
December 24	*Tension*	89.6	Christmas eve
	Anger	87.5	
	Vigor	87.7	
	Fatigue	85.9	
	Confusion	88.4	
December 25	*Tension*	86.0	Christmas
	Vigor	87.5	
	Fatigue	87.7	
December 31	*Tension*	87.2	New Year's Eve
	Fatigue	81.5	

3 Emotion dynamics before removing periodic cycles

Here we show autocorrelation functions and p-values of augmented Dickey-Fuller (ADF) test for emotion dynamics $Z^k(t)$ before removing periodic cycles . For stationary samples, we use p-values are less than 0.01 in Table 2.

Table 2: p-values of ADF test for emotion dynamics $Z^k(t)$ before removing periodic cycles by year.

	Tension	*Depression*	*Anger*	*Vigor*	*Fatigue*	*Confusion*
2006	0.225	0.674	0.495	0.416	0.139	0.707
2007	$< .01$	0.132	0.011	$< .01$	0.213	0.081
2008	$< .01$	0.663	0.023	0.383	0.114	0.942
2009	0.046	0.088	0.067	0.031	0.626	0.012
2010	$< .01$	$< .01$	0.031	0.129	0.233	$< .01$
2011	< 0.043	$< .01$	0.043	0.626	0.375	$< .01$
2012	$< .01$	0.029	0.048	0.804	0.102	0.226
2013	$< .01$	$< .01$	$< .01$	0.030	0.296	$< .01$
2014	$< .01$	0.019	0.066	0.602	0.504	0.404
2015	$< .01$	0.012	0.098	0.384	0.542	0.015

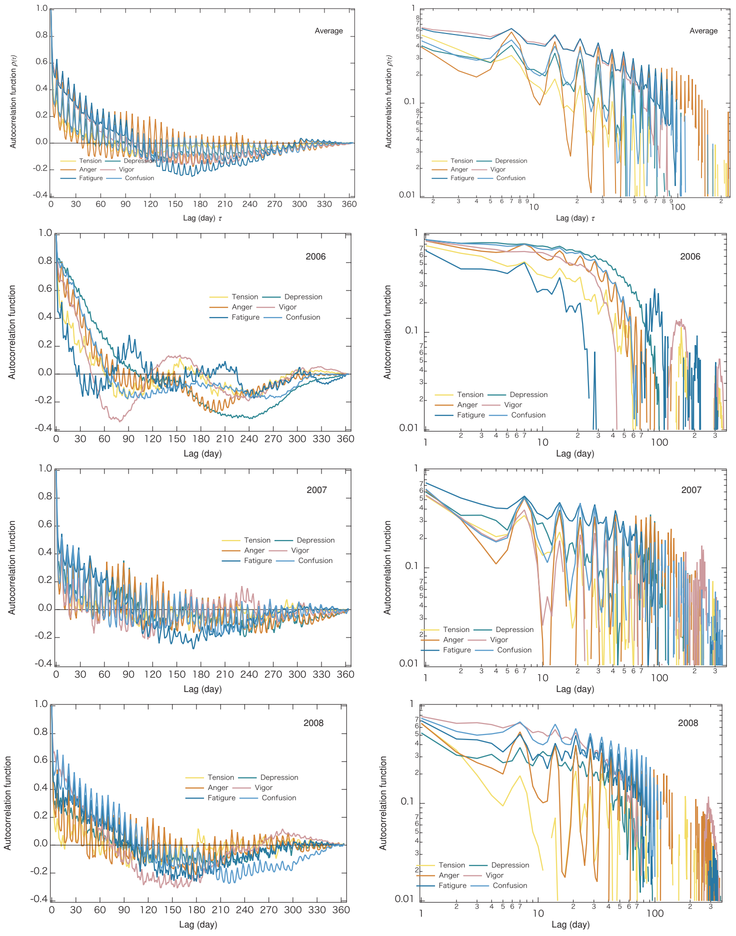

Average
Autocorrelation function ρ(τ)
Lag (day) τ
Tension
Depression
Anger
Vigor
Fatigure
Confusion
2006
2007
2008
Autocorrelation function
Lag (day)

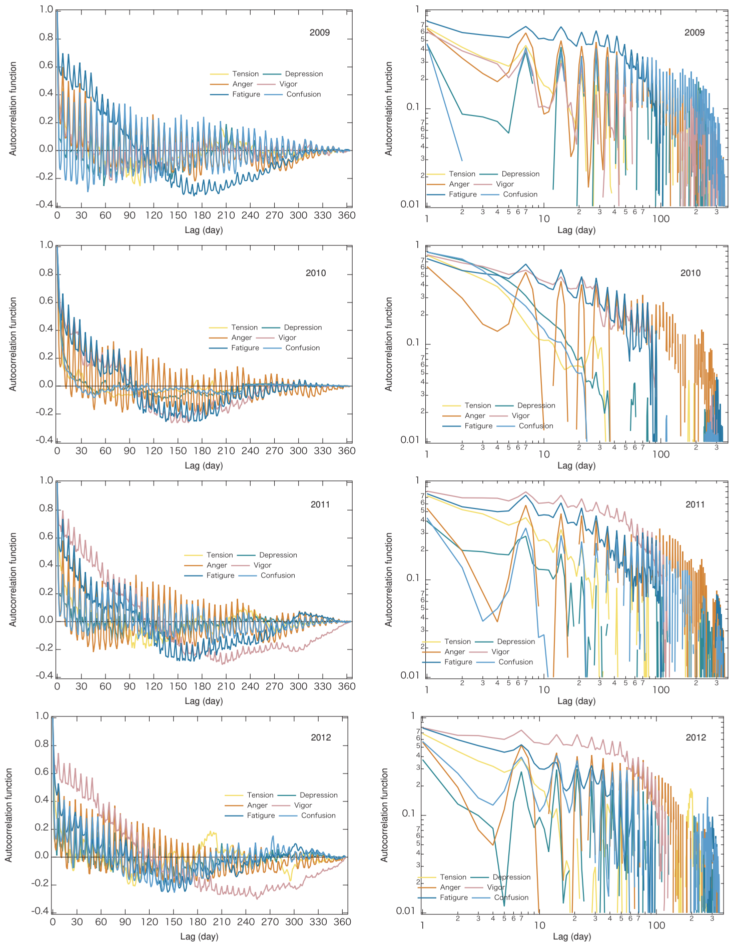
2009
2010
2011
2012
Tension
Anger
Fatigure
Depression
Vigor
Confusion
Autocorrelation function
Lag (day)

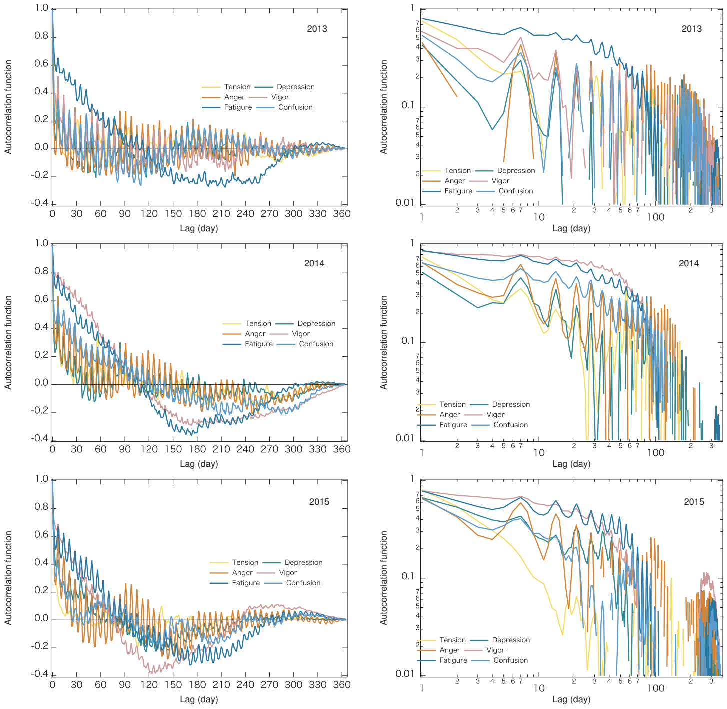

Figure 2: Autocorrelation functions before removing periodic cycles in linear (left) and log-log (right) scale. The averaged autocorrelation function and autocorrelation functions by each year since 2006 are displayed.

4 Emotion dynamics after removing periodic cycles

Here we show monthly emotion dynamics $Z^k(t)$ after removing periodic cycles (Fig. 3). We also show p-values of ADF test (Table 3) and autocorrelation functions (Fig. 4) for emotion dynamics $Z^k(t)$ after removing periodic cycles. For stationary samples, we use p-values are less than 0.01 in Table 3.

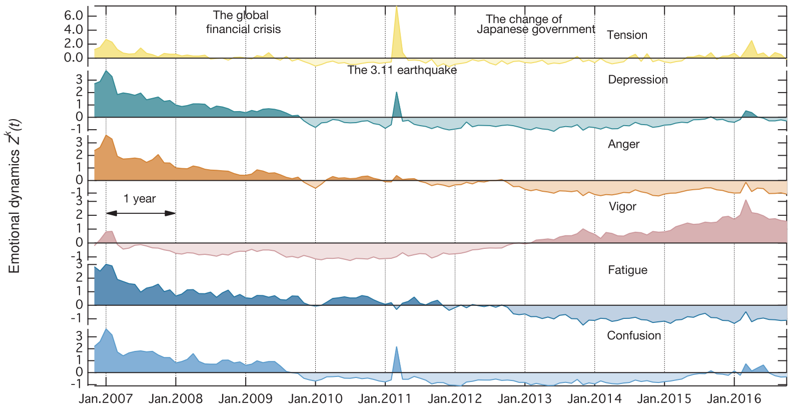

Figure 3: Monthly changes for each emotional dimension after removing all periodic cycles over 10 years. Dashed lines are time series data smoothed by the average of previous six months.

Table 3: p-values of ADF test for emotion dynamics $Z^k(t)$ after removing periodic cycles by year.

	Tension	*Depression*	*Anger*	*Vigor*	*Fatigue*	*Confusion*
2006	$< .01$	0.283	0.437	0.546	0.299	0.661
2007	$< .01$	$< .01$	$< .01$	$< .01$	$< .01$	$< .01$
2008	$< .01$	$< .01$	$< .01$	0.057	$< .01$	0.151
2009	$< .01$	$< .01$	$< .01$	0.036	0.064	$< .01$
2010	$< .01$	$< .01$	$< .01$	$< .01$	$< .01$	$< .01$
2011	$< .01$	$< .01$	$< .01$	0.277	$< .01$	$< .01$
2012	$< .01$	$< .01$	0.540	0.504	0.014	$< .01$
2013	$< .01$	$< .01$	$< .01$	$< .01$	$< .01$	$< .01$
2014	$< .01$	0.048	0.057	0.425	0.023	0.442
2015	$< .01$	$< .01$	0.012	0.219	$< .01$	$< .01$

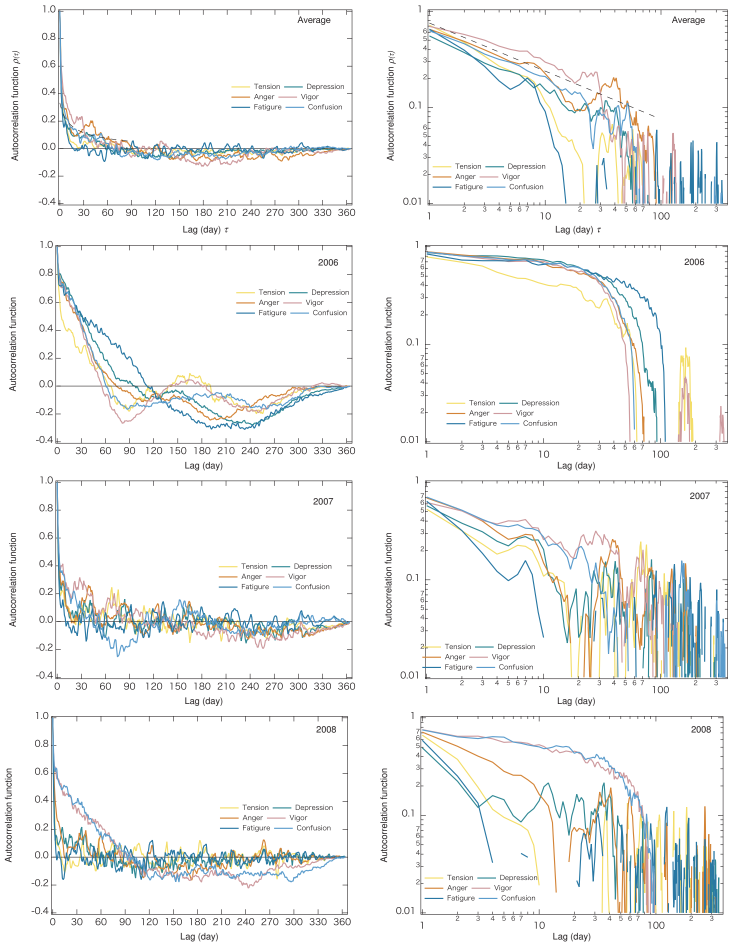
Average
2006
2007
2008
Tension
Depression
Anger
Vigor
Fatigure
Confusion
Autocorrelation function $\rho(\tau)$
Autocorrelation function
Lag (day) τ
Lag (day)

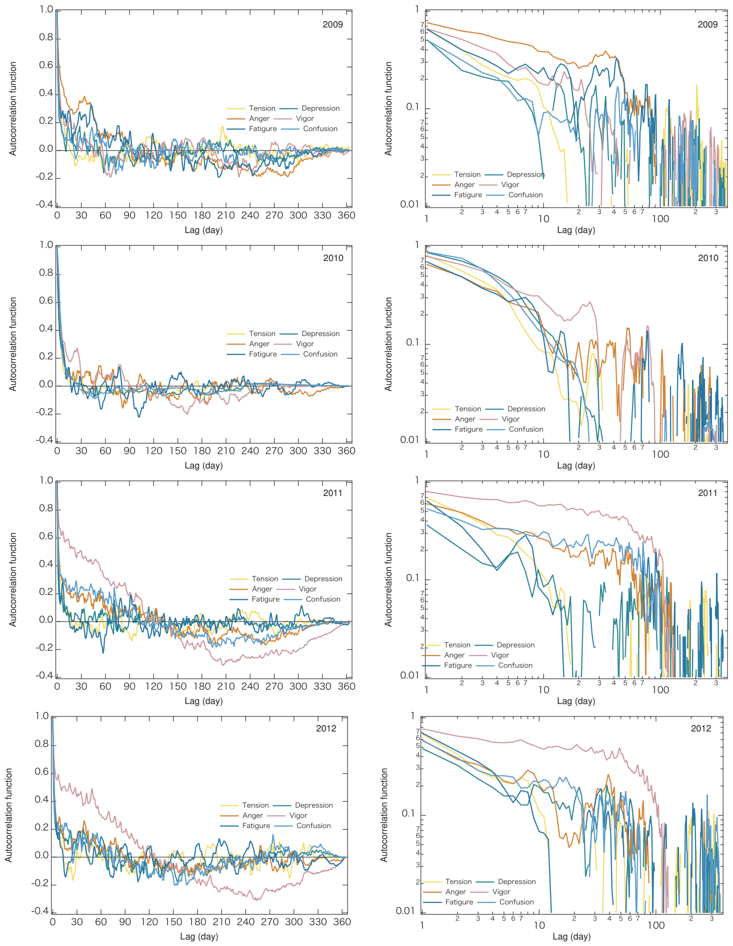
2009
2010
2011
2012
Tension
Depression
Anger
Vigor
Fatigure
Confusion
Autocorrelation function
Lag (day)

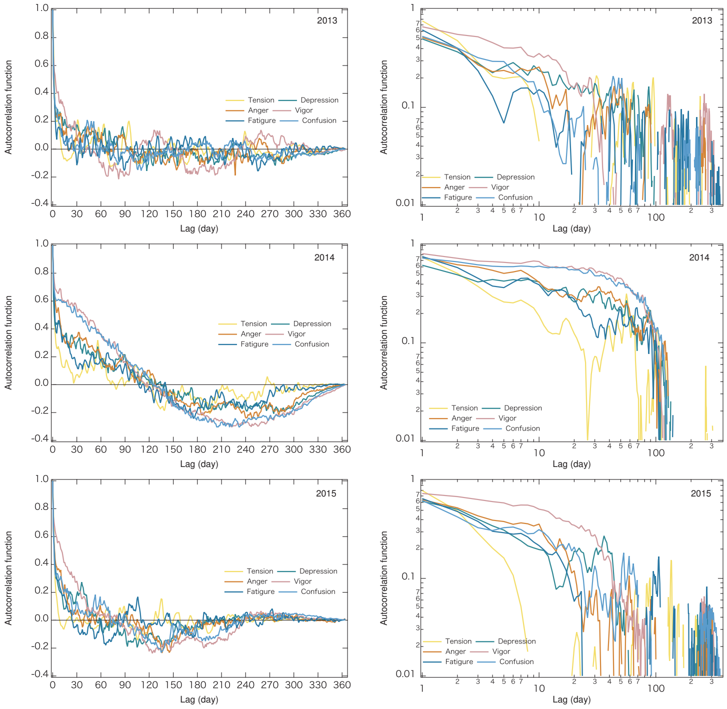

Figure 4: Autocorrelation functions after removing periodic cycles in linear (left) and log-log (right) scale. The averaged autocorrelation function and autocorrelation functions by each year since 2006 are displayed.

5 Results of monthly and daily randomized series

We show autocorrelation functions, power spectrum densities (Fig. 5) of randomized series for monthly and daily basis.

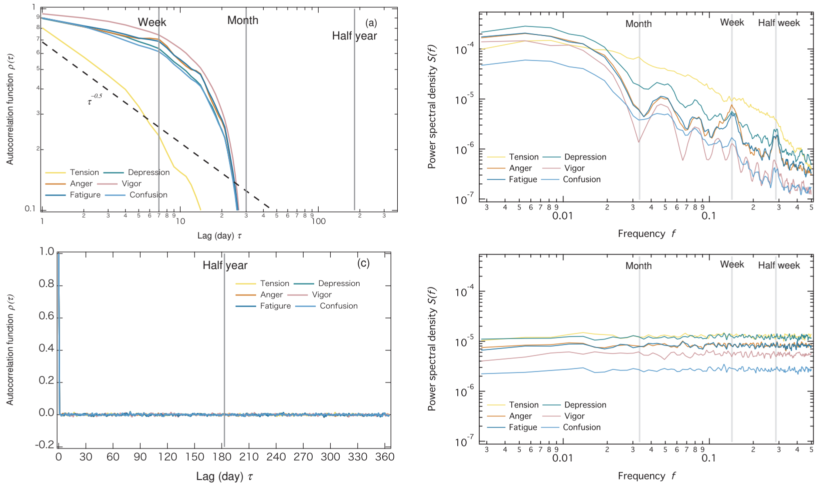

Figure 5: Autocorrelation functions (left) and power spectral densities (right) for randomized series. (a)(b) The results of monthly shuffled series in log-log scale. The results for daily shuffled series in linear (c) and log-log scale (d).

6 Histogram of differences of collective emotion

We show the histograms of daily difference of collective emotion dynamics after removing periodic cycles.

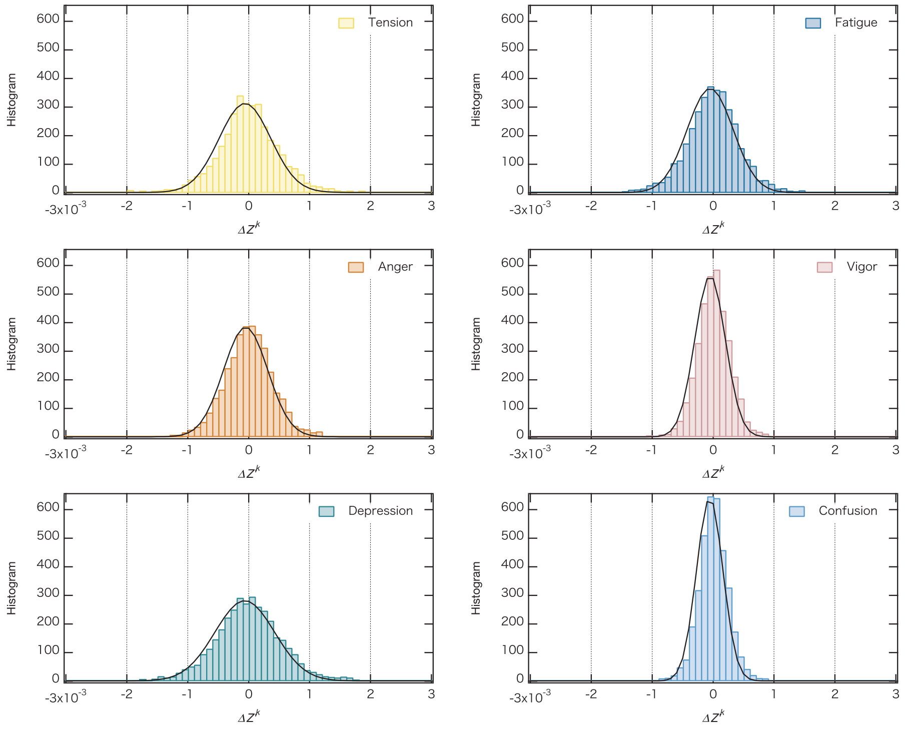

Figure 6: Histograms of daily difference $\Delta Z^k(t)$ of emotions after removing periodic cycles. Each bin size is 1.0×10^{-4} between -3.0×10^{-3} to 3.0×10^{-3}. All solid lines indicate normal distributions.
